# EGCG Protects against 6-OHDA-Induced Neurotoxicity in a Cell Culture Model

**DOI:** 10.1155/2015/843906

**Published:** 2015-12-06

**Authors:** Dan Chen, Anumantha G. Kanthasamy, Manju B. Reddy

**Affiliations:** ^1^Department of Food Sciences and Human Nutrition, Iowa State University, 220 Mackay Hall, Ames, IA 50010, USA; ^2^Department of Biomedical Sciences, Iowa State University, Ames, IA 50010, USA

## Abstract

*Background*. Parkinson's disease (PD) is a progressive neurodegenerative disease that causes severe brain dopamine depletion. Disruption of iron metabolism may be involved in the PD progression.* Objective*. To test the protective effect of (−)-epigallocatechin-3-gallate (EGCG) against 6-hydroxydopamine- (6-OHDA-) induced neurotoxicity by regulating iron metabolism in N27 cells.* Methods*. Protection by EGCG in N27 cells was assessed by SYTOX green assay, MTT, and caspase-3 activity. Iron regulatory gene and protein expression were measured by RT-PCR and Western blotting. Intracellular iron uptake was measured using ^55^Fe. The EGCG protection was further tested in primary mesencephalic dopaminergic neurons by immunocytochemistry.* Results*. EGCG protected against 6-OHDA-induced cell toxicity. 6-OHDA treatment significantly (*p* < 0.05) increased divalent metal transporter-1 (DMT1) and hepcidin and decreased ferroportin 1 (Fpn1) level, whereas pretreatment with EGCG counteracted the effects. The increased ^55^Fe (by 96%, *p* < 0.01) cell uptake confirmed the iron burden by 6-OHDA and was reduced by EGCG by 27% (*p* < 0.05), supporting the DMT1 results. Pretreatment with EGCG and 6-OHDA significantly increased (*p* < 0.0001) TH^+^ cell count (~3-fold) and neurite length (~12-fold) compared to 6-OHDA alone in primary mesencephalic neurons.* Conclusions*. Pretreatment with EGCG protected against 6-OHDA-induced neurotoxicity by regulating genes and proteins involved in brain iron homeostasis, especially modulating hepcidin levels.

## 1. Introduction

Iron plays a crucial role in many physiological functions including DNA synthesis, oxygen transport, and mitochondrial respiration [1–3]. Iron also serves as a cofactor of many enzymes, such as tyrosine hydroxylase, which is involved in brain function. It is important to regulate intracellular iron uptake, transport, and storage to maintain iron homeostasis because both low and high iron levels can be detrimental to brain function. The accumulation of non-protein-bound iron in the cell can induce oxidative damage to cellular organelles by producing free radicals via the Fenton reaction [4], resulting in chronic neurological disorders, including Parkinson's disease (PD) [5, 6].

Divalent metal transporter-1 (DMT1) is one of the proteins responsible for cell iron uptake. DMT1 can be upregulated by neurotoxins, such as 6-hydroxydopamine (6-OHDA), resulting in excess iron influx and neurodegeneration [7–9]. However, it is still unclear whether iron influx and subsequent free radical formation is a primary or a secondary event in the neurodegenerative process of PD. Hepcidin is expressed in several organs, such as the liver, brain, and spinal cord, and is a key iron regulatory protein responsible for normal iron homeostasis. Hepcidin can bind to iron exporter ferroportin 1 (Fpn1), causing its internalization and degradation, thereby promoting iron retention in the cell [10]. Hepcidin expression is influenced by intracellular iron concentration and oxidative stress [11]. Because 6-OHDA has been shown to alter DMT1 expression and induce oxidative stress [7], it is possible that 6-OHDA may exert its neurotoxicity by interrupting iron homeostasis through hepcidin and, consequently, may cause more oxidative damage. It has been shown that 6-OHDA also can release iron from ferritin, which can induce oxidative stress, causing neuronal cell death [12, 13]. However, the detailed mechanisms remain unclear.

Green tea polyphenols have been shown to provide beneficial effects against cancer, inflammation, and neurological disorders. (−)-Epigallocatechin-3-gallate (EGCG) is the most abundant polyphenol in green tea and has been shown to protect against neurotoxicity in several cell culture models due to its capability of scavenging free radicals and chelating iron [14, 15]. Several* in vitro* studies have shown that EGCG reduces 6-OHDA neurotoxicity, but the mechanism of the protection is not clear. We hypothesized that 6-OHDA disrupts brain iron metabolism by altering iron-related proteins and that EGCG administration would normalize these adverse effects. We used immortalized rat mesencephalic dopaminergic neuronal cell line (N27 cells) as well as primary mesencephalic dopaminergic neurons to determine the neuroprotective effect of EGCG against 6-OHDA. We also determined whether EGCG protection of normalizing the disruption of iron metabolism induced by 6-OHDA is by regulation of DMT1, Fpn1, and hepcidin.

## 2. Material and Methods

### 2.1. Reagents

EGCG, 6-OHDA, and thiazolyl blue tetrazolium bromide (MTT) were purchased from Sigma-Aldrich (St. Louis, MO), and SYTOX Green Nucleic Acid Stain was purchased from Molecular Probes (Eugene, OR). Substrate for caspase-3, acetyl-Asp-Glu-Val-Asp-AFC (Ac-DEVD-AFC), was obtained from MP Biomedicals (Solon, OH). The mouse TH^+^ antibody was obtained from Millipore (Temecula, CA); Alexa 680-conjugated anti-mouse secondary antibodies, RPMI-1640 medium, fetal bovine serum, L-glutamine, penicillin, streptomycin and neurobasal medium, B27 supplement, and Dulbecco's modified Eagle's medium (DMEM) were obtained from Invitrogen (Carlsbad, CA). Rabbit anti-rat DMT1 polyclonal antibody (Catalog: NRAMP21A), rabbit anti-rat MTP1 polyclonal antibody (Catalog: MTP11-A), and rabbit anti-rat hepcidin polyclonal antibody (Catalog: HEPC-11A) were purchased from Alpha Diagnostic International (San Antonio, TX), and *β*-actin mouse anti-mouse monoclonal antibody was purchased from Sigma-Aldrich (St. Louis, MO). Secondary goat anti-rabbit IgG-HRP (Catalog: sc-2004) and goat anti-mouse IgG-HRP (Catalog: sc-2005) were obtained from Santa Cruz Biotechnology (Dallas, TX). SuperSignal West Femto chemiluminescent substrate for Western blotting was obtained from Thermo Scientific (Rockford, IL). ^55^Fe was purchased from PerkinElmer (Waltham, MA). Scintanalyzed ScintiVerse BD Cocktail was purchased from Fisher Scientific (Pittsburgh, PA).

### 2.2. Cell Culture

N27 cells were kindly gifted by Dr. Kedar N. Prasad (University of Colorado Health Sciences Center, Denver, CO) and were grown in RPMI-1640 medium containing 10% fetal bovine serum, 2 mM L-glutamine, 50 U penicillin, and 50 *μ*g/mL streptomycin and maintained at 37°C in a humidified atmosphere containing 5% CO_2_ as described in previous studies [16].

We also used primary mesencephalic dopaminergic neuronal cultures to determine the neuroprotective effect of EGCG on TH^+^ cells. All of the procedures involving animal handling were approved by the Institutional Animal Care Use Committee (IACUC) at Iowa State University. Primary cultures were prepared from the ventral mesencephalon of gestational 14-day-old C57 black mice embryos as described previously [17]. Mesencephalic tissues were dissected and kept in ice-cold Ca^2+^-free Hanks' balanced salt solution. Cells then were dissociated in Hanks' balanced salt solution containing trypsin 0.25% EDTA for 30 min at 37°C. Cells were suspended in neurobasal medium with 2% neurobasal supplement (B27) after 10% FBS inactivating enzyme activity in DMEM medium. The cells were then plated at a density of 0.5 × 10^6^ cells on 12 mm coverslips precoated with 1 mg/mL poly-D-lysine. Cell cultures were maintained in 500 *μ*M of L-glutamine, 100 IU/mL of penicillin, and 100 units of streptomycin neurobasal medium and incubated in a humidified CO_2_ incubator (5% CO_2_ and 37°C). Half of the cell media were replaced every 2 days, and assays were conducted using cultures at 7 days. Primary cultures were exposed to 6-OHDA (25 *μ*M) for 24 h in the presence or absence of 2 h of pretreatment with EGCG (1, 10, and 100 *μ*M, resp.). Cells were then fixed for immunocytochemical analysis.

### 2.3. SYTOX Green Assay

N27 cells grown in 24-well plates with 500 *μ*L of RPMI-1640 medium were coincubated with 1 : 1000 SYTOX green dye. To determine the dose-response protective effect of EGCG from 6-OHDA damage, N27 cells were pretreated with EGCG at concentrations of 1, 10, 50, and 100 *μ*M for 2 h, followed by 6-OHDA (100 *μ*M) treatment for 6 h. The assessment of cell death was conducted using SYTOX green nucleic stain as described previously [17]. After each treatment, SYTOX green fluorescence signal was detected using a microplate reader (Bio-Tek microplate reader, VT) at an excitation wavelength of 485 nm and an emission wavelength of 538 nm. The intensity of fluorescence was directly proportional to the number of dead cells, which was monitored by Nikon inverted fluorescence microscope equipment (Model TE-2000 U, Diagnostic Instruments, Sterling Heights, MI).

### 2.4. Cell Viability

The N27 cells were pretreated and cotreated with 100 *μ*M of EGCG followed by 6-OHDA treatment for 6 h, and the cell viability was measured using MTT assay as described previously [18]. Briefly, cells were washed with PBS after treatments and then incubated with serum-free RPMI medium containing 0.25 mg/mL of MTT reagent for 3 h at 37°C. Isopropanol-HCl (0.04 M) solution was added to dissolve intracellular purple formazan, and absorbance was read at 570 nm with a reference wavelength of 630 nm using a microplate reader (Molecular Devices, Sunnyvale, CA) to assess cell viability.

### 2.5. Caspase-3 Activity

Caspase-3 activity was measured to assess cell apoptosis as previously described [19]. Briefly, N27 cells were pretreated with 100 *μ*M of EGCG for 2 h followed by 100 *μ*M 6-OHDA treatment for 6 h. Then, the cells were suspended in lysis buffer (50 mM of Tris-HCl, 1 mM of EDTA, and 10 M of EGTA) containing 10 mM of digitonin for 20 min at 37°C. Supernatants were treated with the fluorogenic substrate Ac-DEVD-AFC for 1 h at 37°C, and fluorescence was measured at excitation at 400 nm and emission at 505 nm using a microplate reader. The activity was measured as fluorescent units per mg protein.

### 2.6. Immunocytochemistry

The primary mesencephalic dopaminergic neurons were used for analysis of TH^+^ cells by using an immunocytochemistry method [16]. Cells were fixed with 4% paraformaldehyde and permeabilized. Nonspecific sites were blocked with 5% normal goat serum containing 0.4% BSA and 0.2% Triton-X 100 in PBS for 20 min. Cells were then incubated with antibodies directed against TH^+^ (1 : 500) overnight at 4°C followed by incubation with Cy3-conjugated (1 : 1000) secondary antibody for 1 h at room temperature. Secondary antibody treatments were followed by incubation with 10 *μ*g/mL of Hoechst 33342 for 3 min at room temperature to stain the nucleus. Then, the coverslips containing cells were washed with PBS, mounted on a slide, and viewed under a Nikon inverted fluorescence microscope; images were captured with a SPOT digital camera (Diagnostic Instruments, Sterling Heights, MI). TH^+^ cell count and neurite processes were measured as described previously [16]. For measurement of TH^+^ cell count, MetaMorph image analysis software was used. Neuronal count and volume were measured on the threshold images using Integrated Morphometry Analysis (IMA). Neuronal process lengths were marked by applying the region and length measurement function in the IMA [20].

### 2.7. Quantitative Real-Time PCR Analyses

The mRNA levels of DMT1, Fpn1, hepcidin, transferrin receptor (TfR), and H-ferritin were quantified using real-time PCR for gene expression analysis as described previously [21, 22]. N27 cells were pretreated with 100 *μ*M of EGCG followed by 25 *μ*M of 6-OHDA for 24 h. We used low concentration of 6-OHDA in these experiments to prevent severe cell death so that we would be able to detect the expression of iron-related proteins. Total RNA was isolated from the cells using Absolutely RNA Miniprep Kits (Stratagene, Santa Clara, CA) and reverse transcribed by High Capacity cDNA Reverse Transcription Kits (Applied Biosystems, Foster City, CA). RT-PCR was performed using the Brilliant SYBR Green QPCR Master Mix Kit and the Mx3000P QPCR System (Stratagene, Santa Clara, CA). The 25 *μ*L PCR reaction mixtures included 1 *μ*L of cDNA (produced by 100 ng of RNA), 12.5 *μ*L of 2x master mix, and 100 nM of each primer (synthesized by Integrated DNA Technologies, Coralville, IA, listed in Table 1). Cycling conditions contained an initial denaturation at 95°C for 10 min followed by 40 cycles of amplification at 95°C for 30 s for denaturation, 60°C for 30 s for annealing, and 72°C for 30 s for extension. Fluorescence was detected during the annealing/extension step of each cycle. The* GAPDH* gene was used as an internal control. A comparative threshold cycle method was used to analyze the data. All reactions were performed in triplicate. Results were compared to the control without any treatment.

### 2.8. Western Blot Analyses

DMT1, hepcidin, and Fpn1 protein expressions were evaluated by Western blot assay. N27 cells were pretreated with 100 *μ*M of EGCG followed by 25 *μ*M of 6-OHDA for 24 h. Then, the cells were washed with cold PBS, homogenized in RIPA buffer containing 1% Triton X-100, 0.1% SDS, 1 mM of phenylmethanesulfonyl fluoride (PMSF), and protease inhibitors (1 mg/mL each of pepstatin, aprotinin, and leupeptin), and then sonicated on ice. The supernatant was collected after centrifugation at 10,000 g for 15 min at 4°C, and protein concentration was determined. Forty micrograms of protein extract was mixed with an equal volume of 5x sample buffer (0.35 M Tris-HCl, 10% SDS, 30% glycerol, and 0.012% bromophenol blue) and loaded onto a 12% SDS-polyacrylamide gel or 15% urea gel, electrophoresed, and then transferred to a nitrocellulose membrane (Bio-Rad, Hercules, CA). The membrane was blocked with 5% nonfat dry milk in TBS with Tween and then incubated with rabbit anti-rat DMT1 with IRE polyclonal antibody (1 : 200), rabbit anti-rat Fpn1 polyclonal antibody (1 : 500), or rabbit anti-rat hepcidin polyclonal antibody (1 : 500), followed by horseradish peroxidase-conjugated goat anti-rat IgG antibody (1 : 3000). *β*-actin (1 : 2000) was used to normalize protein loading. All Western blot assays were performed in triplicate in two separate experiments, and the band densities were compared to the control without any treatment.

### 2.9. Cellular Iron Uptake

N27 cells were grown in 12-well plates and exposed to 25 *μ*M of 6-OHDA after 2 h pretreatment with 100 *μ*M of EGCG. Ferric chloride (0.5 *μ*Ci/mL ^55^Fe) was added to the cell medium and incubated at 37°C for 24 h. Cells were then washed twice with cold 1x PBS, lysed in 250 *μ*L of RIPA buffer, and centrifuged at 10,000 g for 10 min at 4°C. The ^55^Fe radioactivity in the supernatant was measured in a scintillation counter premixed with 10 mL scintillation cocktail. Cell uptake was measured based on the fraction of radioactivity found in the cell lysate in relation to the initially added amount. The experiments were performed in duplicate, and the ^55^Fe uptake was represented as counts per minute normalized to cell protein concentration.

### 2.10. Statistical Analyses

Data were analyzed with Prism 4.0 software (Graph Software, San Diego, CA). All values are expressed as the mean ± SEM and represented as a percentage of the respective controls. ANOVA with Tukey's multiple comparison tests was used to detect the differences among the treatments. Student's *t*-test was used to compare the differences in iron-related gene expression between 6-OHDA with EGCG pretreatment and 6-OHDA treatment alone groups. The mean differences were considered significant at *p* ≤ 0.05.

## 3. Results

### 3.1. Effects of 6-OHDA and EGCG on Cell Death

**Figure 1:**
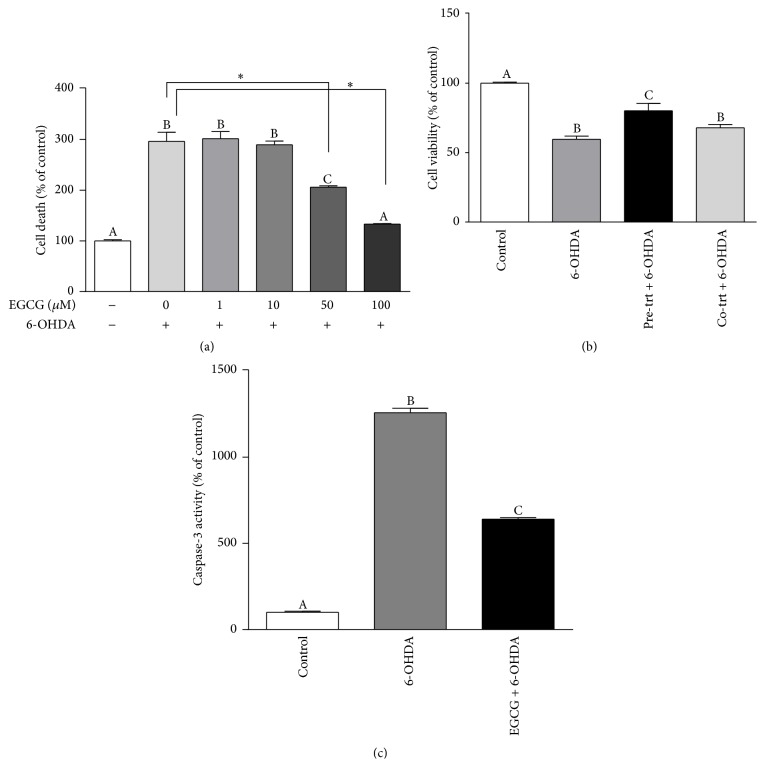
Effect of 6-OHDA and EGCG on N27 cell death, cell viability, and cell apoptosis. Data are represented as % of control (no treatment). Cell death (a) was determined by SYTOX green assay, and values are mean ± SEM, *n* = 8. Cells were pretreated with EGCG for 2 h followed by 100 *μ*M 6-OHDA treatment for 6 h. Bars without common letters differ. Cell viability (b) was evaluated by MTT assay. 6-OHDA, 100 *μ*moles/L for 6 h; EGCG, 100 *μ*moles/L. Pre-trt EGCG: pretreatment for 2 h; Co-trt EGCG: cotreatment with 6-OHDA. Values are mean ± SEM, *n* = 6. Cell apoptosis (c) was measured as caspase-3 activity. 6-OHDA (100 *μ*M) for 6 h following EGCG (100 *μ*M) pretreatment for 2 h. Values are mean ± SEM, *n* = 3. Labeled bars without a common letter differ. ^*∗*^ *p* < 0.001.

Based on the SYTOX green assay, EGCG protected against 6-OHD-induced cell death in a dose-response manner (Figure 1(a)). 6-OHDA significantly increased cell death by ~3-fold (*p* < 0.0001) compared to the control. Pretreatment with EGCG at a concentration of 50 and 100 *μ*M for 2 h decreased cell death by 31% (*p* < 0.0001) and 55% (*p* < 0.0001), respectively. Cell death with 100 *μ*M EGCG pretreatment was not significantly different from the control but was significantly lower than for the 6-OHDA alone. No protection for cell death was found at low concentrations of EGCG (1 and 10 *μ*M).

### 3.2. EGCG Protected against 6-OHDA-Induced Decreased Cell Viability

As shown in Figure 1(b), cell viability was decreased to 59% (*p* < 0.001) after 6 h of treatment with 6-OHDA, but EGCG pretreatment for 2 h significantly protected against this toxicity by 21% (*p* < 0.01). In contrast, no protection for cell viability was found with the EGCG cotreatment group, suggesting that EGCG does not provide a neurorescue effect.

### 3.3. EGCG Protected against 6-OHDA-Induced Cell Apoptosis

Cell apoptosis, as measured by caspase-3 activity (Figure 1(c)), was determined only with pretreatment, not cotreatment, of ECCG as it did not show a neurorescue effect in the cell viability experiments. Compared to control, 6-OHDA significantly increased caspase-3 activity by ~12-fold (*p* < 0.0001), whereas pretreatment with EGCG for 2 h decreased the caspase-3 activity by 49% (*p* < 0.0001) compared to 6-OHDA treatment.

### 3.4. EGCG Decreased TH^+^ Neuronal Loss from 6-OHDA in Primary Cultures

**Figure 2:**
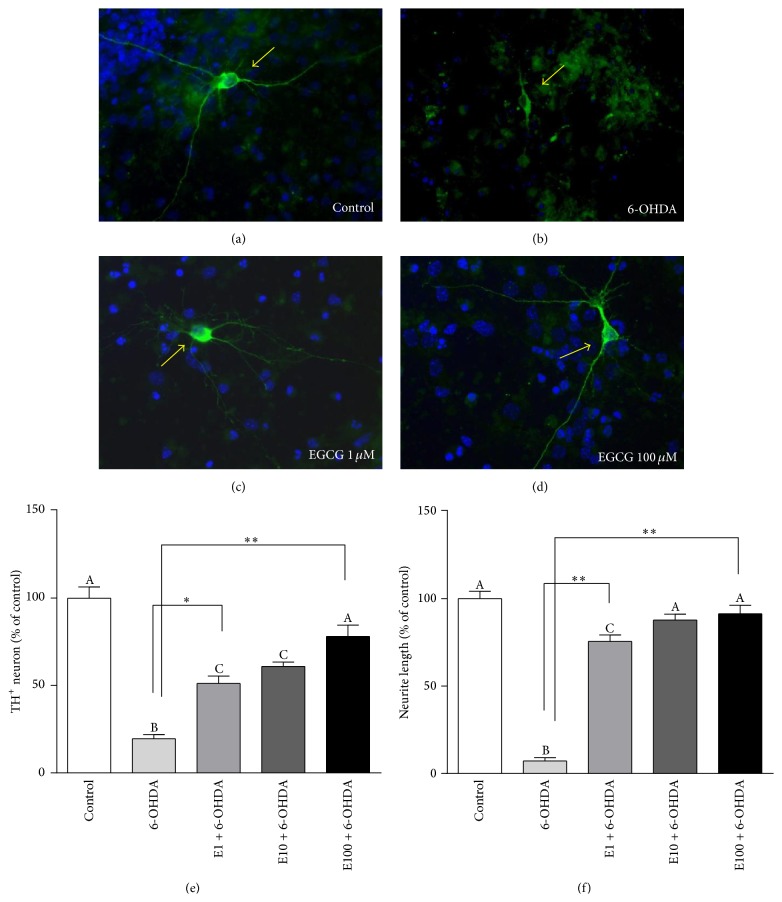
Prevention of TH^+^ neuronal loss by EGCG induced by 6-OHDA in primary cultures. Cells were pretreated with EGCG (1, 10, and 100 *μ*M) for 2 h followed by 6-OHDA (10 *μ*M) for 24 h. Immunocytochemistry was used to measure the TH^+^ cells (arrow, (a), (b), (c), and (d)). Quantification data on TH^+^ number (e) and neurite length (f). Values are mean ± SEM, *n* = 3. Bars without common letters differ. ^*∗*^ *p* < 0.01; ^*∗∗*^ *p* < 0.0001.

Treatment with 6-OHDA for 24 h induced ~80% (*p* < 0.0001) loss of TH^+^ cell count (Figure 2(e)), compared to the control. Pretreatment with EGCG (1, 10, and 100 *μ*M) for 2 h significantly increased TH^+^ cell count by 155% (*p* < 0.01), 205% (*p* < 0.001), and 290% (*p* < 0.0001), respectively (Figure 2(e)). EGCG at 100 *μ*M concentration completely protected TH^+^ cell loss, as shown by the nonsignificant difference when compared to the control. The average lengths of TH^+^ neuronal processes in EGCG-pretreated cells were significantly longer than those treated with only 6-OHDA. We found that 6-OHDA significantly decreased the TH^+^ neurite length by 93% (*p* < 0.0001), whereas pretreatment with EGCG (1, 10, and 100 *μ*M) for 2 h significantly increased the neurite length by ~10-fold (*p* < 0.0001), 12-fold (*p* < 0.0001), and 12-fold (*p* < 0.0001), respectively (Figure 2(f)). In addition, Hoechst staining was used to verify the cell viability. 6-OHDA significantly decreased Hoechst activity, whereas pretreatment with EGCG (1, 10, or 100 *μ*M) increased Hoechst staining (data not shown), suggesting that the cell viability in the primary culture increased with EGCG. Overall, the protection of EGCG against 6-OHDA toxicity was shown to be effective, even in the primary cultures.

### 3.5. EGCG Altered the Effect of 6-OHDA on the mRNA Expression of Iron-Related Genes

As shown in Table 2, 6-OHDA at 25 *μ*M for 24 h significantly increased the mRNA expression of DMT1 + IRE, hepcidin, TfR2, and H-ferritin and decreased the mRNA expression of Fpn1 and TfR1. However, EGCG counteracted the effect by decreasing the mRNA expression of DMT1 + IRE by 60% (*p* < 0.001), hepcidin by 54% (*p* < 0.05), H-ferritin by 53% (*p* < 0.05), and TfR2 by 27% (*p* < 0.05) while increasing the mRNA expression of Fpn1 by 70% (*p* < 0.01) and TfR1 by 96% (*p* < 0.05), compared to treatment with only 6-OHDA, suggesting the reduced iron burden in the cell.

### 3.6. EGCG Altered the 6-OHDA Effect on Iron-Related Protein Expressions

**Figure 3:**
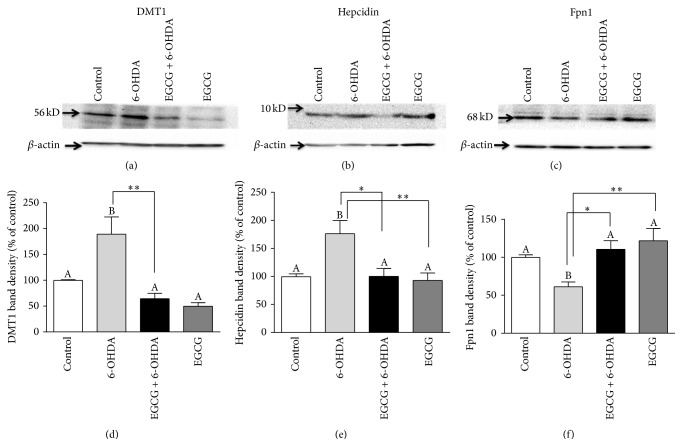
Western blot analysis of the effect of 6-OHDA and EGCG on iron-related protein expression in N27 cells, DMT1 (+IRE) (a), hepcidin (b), and Fpn1 (c), which were normalized by *β*-actin. Cells were treated with EGCG (100 *μ*M) for 2 h prior to treatment with 6-OHDA (25 *μ*M) for 24 h. Quantitative data for band intensity are shown in bottom panels, (d), (e), and (f), respectively. Values are mean ± SEM, *n* = 3. Bars without a common letter differ. ^*∗*^ *p* < 0.05; ^*∗∗*^ *p* < 0.01.

Similar to the gene expression results, 6-OHDA significantly increased protein expression of DMT1 + IRE by 2-fold (*p* < 0.05) and hepcidin by 77% (*p* < 0.05) and decreased expression of Fpn1 by 61% (*p* < 0.0001) compared to the control. Pretreatment with EGCG normalized these effects by decreasing DMT1 + IRE by 66% (*p* < 0.01) and hepcidin by 43% (*p* < 0.05) and increasing Fpn1 by 82% (*p* < 0.01) compared to treatment with 6-OHDA alone (Figure 3).

### 3.7. EGCG Reduced Iron Burden Induced by 6-OHDA

**Figure 4:**
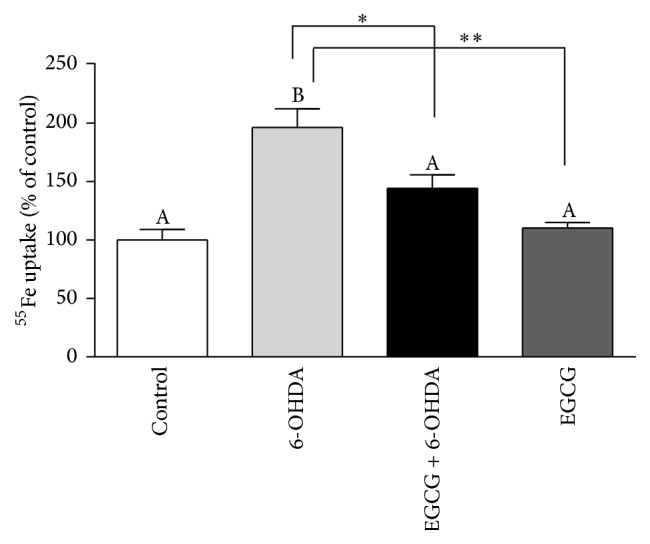
^55^Fe iron uptake by the N27 cell. Cells were treated with EGCG (100 *μ*M) for 2 h followed by 6-OHDA (25 *μ*M) for 24 h. Percentage of ^55^Fe uptake was calculated by measuring the fraction of radioactivity in the cell lysates in relation to initial added radioactivity. Values (mean ± SEM, *n* = 3) are expressed as percentage of controls after normalizing with protein concentration. Bar means without a common letter differ. ^*∗*^ *p* < 0.05; ^*∗∗*^ *p* < 0.01.

As shown in Figure 4, 6-OHDA significantly increased ^55^Fe uptake by 96% (*p* < 0.01). Pretreatment with EGCG significantly decreased cellular ^55^Fe by 27% (*p* < 0.05) compared to treatment with 6-OHDA alone, supporting the results of mRNA and protein expression.

## 4. Discussion

Neurotoxins, such as 6-OHDA, have been widely used to study PD in* in vitro* and* in vivo* studies [7, 23, 24] because of their ability to induce oxidative stress and cause mitochondrial dysfunction. High doses of 6-OHDA are commonly used for studying short-term and acute pathological damage. A previous study showed that 100 *μ*M of 6-OHDA for 6 h caused complete loss of cells [25], but in our study, at the same concentration, 6-OHDA decreased cell viability by only ~40% and increased N27 cell death by ~3-fold and cell apoptosis by ~12-fold. We used a relatively lower dose of 6-OHDA (25 *μ*M) to determine its regulatory effect on iron-related mRNA and protein expression as well as on intracellular iron uptake, given that a lower dose of 6-OHDA favors better understanding of the mechanisms of neurodegeneration before the complete loss of cells [26]. For the primary cell cultures, lower concentrations of 6-OHDA (<50 *μ*M) were mostly used due to the high sensitivity of primary neurons [27, 28], because a higher concentration of 6-OHDA (200 *μ*M) showed complete death in rat primary nigral culture [29]. In this study, we used 10 *μ*M of 6-OHDA to show the significant TH^+^ cell loss after 24 h of treatment. Overall, the dose of 6-OHDA used in different studies depends on the cell type and the purpose of the study.

Several pathways, such as mitochondrial dysfunction, oxidative stress, and inflammation, have already been proposed to explain 6-OHDA-induced neurotoxicity. It also has been reported that 6-OHDA can release iron from ferritin and alter DMT1 expression, suggesting it might exert its toxicity by interrupting iron homeostasis by increasing intracellular iron concentration and, in turn, inducing oxidative stress [30, 31]. In our study, we also found that 6-OHDA altered iron metabolism by altering iron regulatory gene and protein expression. We showed that 6-OHDA increased DMT1 + IRE and hepcidin expression and decreased Fpn1 expression, suggesting high cellular iron uptake and low iron release causing cellular iron burden. The increase in ferritin and transferrin receptor 2 (TfR2) with 6-OHDA treatment supports the hypothesis of increased cellular iron concentrations. The radioactive iron uptake data also supports the DMT1 results by showing increased iron uptake with 6-OHDA.

Our results support previous studies that showed the upregulation of DMT1 by 6-OHDA in an IRE/IRP-dependent manner, indicating that 6-OHDA may affect the activity of IRPs and increase the translation and stabilization of DMT1 mRNA [32, 33]. Because we found that 6-OHDA did not alter the expression of DMT1 without IRE (data not shown), we can speculate that DMT1 regulation by 6-OHDA may be through IRP. The increase of DMT1 might have caused higher iron influx, leading to high oxidative stress.

Hepcidin, a key iron regulator, is mainly involved in regulating iron homeostasis and can be upregulated by increased iron influx and high oxidative stress [34, 35]. TfR2 has been shown to be positively correlated with hepcidin expression [36, 37], and our results showing increased TfR2 and hepcidin expression support that relationship. Hepcidin can bind to Fpn1 and induce its internalization and degradation, decreasing iron export. In our study, we showed that 6-OHDA significantly increased hepcidin and suppressed Fpn1 expression, which may result in higher intracellular iron retention. Although we do not have data to show a decrease in iron release to support the Fpn1 results, we can postulate about that mechanism based on the upregulation of hepcidin. Mixed results have been reported on the effect of neurotoxins on Fpn1. Decreased Fpn1 and increased excess iron were reported in an earlier study [38, 39]. On the contrary, neurotoxins also were reported to upregulate Fpn1 expression in astrocytes and SH-SY5Y cells [39, 40]. The difference in Fpn1 regulation may depend on cell type and the nature of the neurotoxins.

Given that alteration of iron level in the brain has been linked to several neurological disorders, maintaining normal iron homeostasis seems to be an ideal strategy for neuroprotection [41]. As an iron chelator and antioxidant, EGCG has been reported in numerous studies to protect against neurotoxicity [42–44]. In this study, EGCG protected N27 cells against 6-OHDA-induced cell death. The EGCG pretreatment method was shown to be more effective than cotreatment, suggesting that it offers neuroprotection rather than a neurorescue effect. Although EGCG at lower concentrations (10–100 *μ*M) exerted protection in both N27 cells and primary cell cultures, the concentration at 100 *μ*M showed the best protective effect, whereas a higher dose (>200 *μ*M) was found to be toxic (data not shown). The 8 h treatment of EGCG (100 *μ*M) on N27 cells reduced ROS level (*p* < 0.01) compared with control (unpublished data), indicating this level of EGCG did not have toxic effect. This finding matches the bell-shaped pattern of EGCG, typical of antioxidants, showing neuroprotection at low concentrations and a prooxidant effect at higher concentrations [14, 15]. Various doses of EGCG (10–400 *μ*M) also were reported to have a protective effect in other cell models [45, 46]. We used 100 *μ*M of EGCG, which is higher than what one might expect under normal physiological conditions, because low doses had been shown to be less effective. This higher dose may not be achieved with a human diet, but it is possible with dietary supplements.

Several proposed mechanisms may be attributed to the neuroprotection capabilities of EGCG. First, it may regulate iron-related proteins and maintain normal iron homeostasis [47]. In this study, we showed that pretreatment by EGCG counteracted the adverse effect of 6-OHDA on iron-related proteins, leading to less intracellular iron accumulation. The decreased iron accumulation with EGCG pretreatment was supported by our ^55^Fe uptake data, which showed a 27% decrease in iron burden compared with 6-OHDA treatment alone. Second, EGCG may chelate iron, with the complex not being taken up by the cell, reducing the iron-induced reactive oxygen species generation, which we have shown in another study (unpublished results). Third, EGCG also may enhance the antioxidant system by upregulating antioxidant enzymes [14, 48] and scavenging the free radicals directly. The decreased oxidative stress may also affect the iron homeostasis directly or indirectly. EGCG may also suppress inflammatory activity, which may indirectly affect oxidative stress and hepcidin and reduce the iron burden.

## 5. Conclusions

In conclusion, it was shown that EGCG has the potential to prevent neurotoxicity. In this study, we did not see a neurorescue effect of EGCG, because 6-OHDA might have caused acute toxic effect in the* in vitro* condition. Future studies are needed using a lower dosage in animal models. Human studies on PD patients also would provide data to study the effect of EGCG under physiological conditions.

## Figures and Tables

**Table 1:** Effect of 6-OHDA and EGCG on the iron-related gene expression (mean ± SEM, *n* = 3–6).

	6-OHDA	EGCG + 6-OHDA	*p*
DMT1 + IRE	2.3 ± 0.2^*∗*^	0.9 ± 0.0^ns^	<0.001
Hepcidin	1.7 ± 0.1^*∗*^	0.8 ± 0.2^ns^	<0.01
Fpn1	0.7 ± 0.0^*∗*^	1.2 ± 0.2^ns^	<0.05
H-ferritin	2.4 ± 0.4^*∗*^	1.1 ± 0.2^ns^	<0.05
TfR1	0.7 ± 0.0^*∗*^	1.3 ± 0.1^ns^	<0.01
TfR2	1.5 ± 0.1^*∗*^	1.1 ± 0.1^ns^	<0.05

N27 cells were treated with 25 *µ*M of 6-OHDA for 24 h following pretreatment by EGCG 100 *µ*M for 2 h. The values are ratios compared to the control after individual mRNA values were normalized to *GAPDH* expression. *p* values indicate the difference between the 6-OHDA and the EGCG + 6-OHDA treatment groups.

^*∗*^ *p* < 0.05; ns: not significant compared to control.

Differences were determined using Student's *t*-test.

**Table 2:** Summary of the primers for iron-related gene expression.

Hepcidin Forward	GAA GGC AAG ATG GCA CTA AGC A
Hepcidin Reverse	TCT CGT CTG TTG CCG GAG ATA G
Fpn1 Forward	CCA CCT GTG CCT CCC AGA T
Fpn1 Reverse	CCC ATG CCA GCC AAA AAT AC
DMT1 + IRE Forward	CAG TGC TCT GTA CGT AAC CTG TAA GC
DMT1 + IRE Reverse	CGC AGA AGA ACG AGG ACC AA
H-ferritin Forward	GCC CTG AAG AAC TTT GCC AAA T
H-ferritin Reverse	TGC AGG AAG ATT CGT CCA CCT
TfR1 Forward	CTA GTA TCT TGA GGT GGG AGG AAG AG
TfR1 Reverse	GAG AAT CCC AGT GAG GGT CAG A
TFR2 Forward	AGC TGG GAC GGA GGT GAC TT
TFR2 Reverse	TCC AGG CTC ACA TAC ACG ACA G
GAPDH Forward	CCT GGA GAA ACC TGC CAA GTA T
GAPDH Reverse	AGC CCA GGA TGC CCT TTA GT

All the primers were synthesized by Integrated DNA Technologies.
